# Delayed Effects of Different Velocity Loss-Based Resistance Training on Autonomic Regulation, Sleep Quality and Muscle Soreness

**DOI:** 10.5114/jhk/189703

**Published:** 2024-12-06

**Authors:** Juan P. Medellín Ruiz, Oriol Abellán-Aynés, Diana P. García, Luis M. Martínez-Aranda

**Affiliations:** 1 Faculty of Sport, Catholic University of Murcia (UCAM), Murcia, Spain.; 2 Department of Health Sciences, Public University of Navarre (UPNA), Pamplona, Spain.; 3 Physical Activity and Culture Formation (Training) Center - Capital District Regional, National Learning Service (SENA), Bogota D.C., Colombia.; 4 Physical and Sports Performance Research Centre, Faculty of Sports Sciences, Pablo de Olavide University, Seville, Spain.; 5 SEJ-680: Science-Based Training (SBT) research group, Pablo de Olavide University, Seville, Spain.

**Keywords:** resistance training, autonomic nervous system, velocity-based training, physiologic monitoring, 2-point method

## Abstract

Resistance training has been shown to be a stressor factor on the autonomic nervous system, and these changes can be detected by heart rate variability (HRV) analysis. The aim of the present study was to evaluate the delayed effects of velocity loss-based resistance training strategies on heart rate variability (HRV), the sleep quality index (SQI) and delayed onset muscle soreness (DOMS). Fourteen men performed daily recordings. After a baseline period of 14 days of no training, they performed one session per week of resistance training focused on lower body exercise (squats) based on movement velocity. Three resistance training sessions composed of four sets up to 10%, 20% and 40% of velocity loss were performed each week of the study. Statistically significant changes (p < 0.05) after 24 hours of training were found in DOMS, and HRV variables, specifically in RR intervals (RR), root mean square of successive differences of RR intervals (RMSSD), and the percentage of successive RR intervals that differed by more than 50 ms (pNN50), between 40% of velocity movement loss and the rest of conditions. We can conclude that greater losses of execution velocity may result in greater internal load stimuli according to the autonomic modulation measured by HRV. RR, RMSSD and pNN50 seem to be the most sensitive indicators of HRV to fatigue produced by resistance training. This research opens the door to the study of HRV behavior related to resistance training. New research possibilities are raised by measuring the effect of guiding resistance training by means of HRV behavior.

## Introduction

The individual response to training has been proposed as one of the principles of this practice, since not all participants respond in the same way to training loads. In fact, the correlation between training stimulus and the corresponding physiological responses has been shown to be highly individual (Roos et al., 2013). Thus, a correct training load is a key factor when optimizing this response. Likewise, an adequate recovery period will cause a controlled loss of homeostasis and, therefore, initiating adaptive processes, that is known as the Selye’s General Adaptation Syndrome (Cunanan et al., 2018) or the principle of recovery. Finally, to maintain the state of organic adaptation, the principle of progressive loads indicates that the training stimulus must be modified based on intensity, volume, frequency and density; and these changes will maintain a state of fatigue that must be controlled through recovery strategies, avoiding a diminished functional capacity at key moments (Halson, 2014) that can affect optimal performance.

Greater control of the training process using new tools for monitoring the athlete's recovery status leads to an increasingly effective preparation process, especially in high-performance sports, where the results can be defined by a very small margin. The analysis of the state of the autonomic nervous system (ANS) by means of heart rate variability (HRV) has been commonly used as a tool to manage the training load (Achten and Jeukendrup, 2003) and to endurance training prescription (Carrasco-Poyatos et al., 2022).

The physiological basis to justify the use of HRV as a tool to analyze and to guide training is because the ANS describes the automatic regulation of body functions from sympathetic and parasympathetic nerves (Aubert et al., 2003). The ANS response to the execution of moderate physical exercise occurs with the decrease of the vagal tone and the increase of the sympathetic one; therefore, once the physical activity is finished, there is a rapid restoration of the parasympathetic function to the basal state (Palak et al., 2013). However, very intense or prolonged training loads reverse the ANS reaction, giving predominance to the post-exercise sympathetic activity, this being an unfavorable reaction to the vegetative activity of the body (Palak et al., 2013).

Given the control of the ANS in the heart rhythm, the study of HRV has been developed as a function of the time elapsed between each RR interval, allowing evidence of co-regulation (neuromodulation) of cardiac function by the sympathetic and parasympathetic nerves (Yanlin et al., 2020). Currently, the monitoring of HRV is used as a method for evaluating the adaptation to exercise (Buchheit et al., 2010), being its application in training a marker of the internal load (Bourdon et al., 2017). Mainly, the intensity over the volume has been proposed as the component of the load that most affects ANS regulation (Yanlin et al., 2020); thus, HRV becomes a useful marker for long-term monitoring (Roos et al., 2013). In addition, and taking into account that the principle of supercompensation training demands an adequate recovery to achieve an increase in sports performance in an orderly and systematic process, HRV is useful to review the acute and chronic responses of the organism, being probably sensitive to both positive and negative adaptations (Buchheit, 2014); and, this way, it is possible to adjust training loads individually on a daily basis.

Moreover, it has recently been identified that aerobic exercise prescription guided by HRV is effective (Chamera et al., 2023; Düking et al., 2021; Granero-Gallegos et al., 2020; Manresa-Rocamora et al., 2021; Medellín Ruiz et al., 2020). However, only a fraction of the available literature has focused on resistance training. First, it was proposed that after resistance exercise (8 exercises, 3 sets and 10 RM and 90 s of rest between sets) there is a greater vagal suppression of the ANS versus endurance exercise (Heffernan et al., 2006). Kingsley and Figueroa (2014) reviewed the acute effects of resistance exercise (4–11 exercises, 1–5 sets, 30–100% 1RM, 1–20 repetitions and 45–120-s rest intervals) on HRV, finding a prolonged decrease in vagal modulation in healthy young adults. Later, an update confirmed that 30 min after a resistance training session (1–10 exercises, 1–40 sets, 50–100% 1RM, 1–34 repetitions and 30–720-s rest intervals), a general autonomic decrease, withdrawal of cardiac parasympathetic modulation, and activation of cardiac sympathetic modulation were observed, and some components of the training load may also affect or attenuate these modifications (Marasingha-Arachchige et al., 2020). Nevertheless, previous research does not show how long these changes can be maintained. It has been reported that 24 h after different types of resistance training (4 exercises, 2–4 sets, 50–90% 1RM, maximum repetitions and 45–180-s rest intervals), HRV recovers completely (Formalioni et al., 2020), but more studies are needed to support this statement. In the spirit of further investigating the possibility that HRV, DOMS and quality of sleep may reflect the level of fatigue following resistance training, the purpose of this study was to evaluate the delayed effects 24 and 48 h after different velocity loss-based resistance training strategies on HRV, DOMS and quality of sleep. It was hypothesized that HRV, DOMS and quality of sleep would decrease as an effect of a high internal load caused by a demanding training session.

## Methods

### Participants

**Figure 1:**
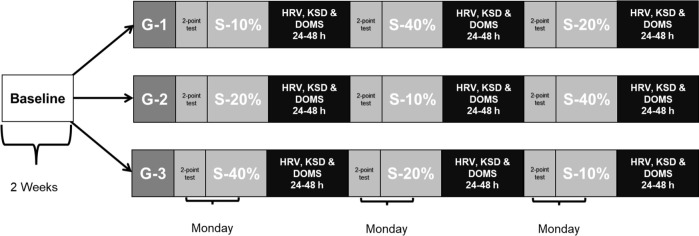
Experimental design.

Fourteen physically active men participated in this study (age: 22.4 ± 2.1 years; body height: 171.9 ± 5.2 cm; body mass: 66.2 ± 6.9 kg). The inclusion criteria were: being over 18 years, being free of musculoskeletal injuries or illnesses that could impede proper physical performance, and not to perform additional training. Participants performed three familiarization sessions of HRV measurements, Karolinska Sleep Diary (KSD) questionnaire procedures, and training methodology based on an eccentric phase at controlled velocity, and a concentric phase at maximum velocity. During the following two weeks, participants completed a baseline period, recording the behavior of HRV, KSD and delayed onset muscle soreness (DOMS) on a daily basis, obtaining the mean normal value when the organism was not under significant fatigue conditions. Then, participants were divided into three groups, and each group performed in random order one type of resistance training session once a week (Figure 1). A standardized warm-up protocol was performed, and this included joint mobility and several repetitions of the specific exercise with low loads. Every session was performed in the morning, under constant environmental conditions (16–18ºC and ~70% humidity). In addition, the control criteria for the participants were to maintain regular diet, hydration and rest, and not to ingest caffeine or alcohol. In the same way, any additional external training sessions were not allowed.

All participants signed an informed consent form before starting the study, and this was approved by the Catholic San Antonio University’s Ethic Committee (approval code: N° 7610-CE52003; approval date: 29 May 2020). Likewise, the study was conducted in accordance with the recommendations of the Declaration of Helsinki.

### Measures

In order to avoid alterations that could affect the rest period and, therefore, the results, the KSD was used to obtain the sleep quality index (SQI), calculated as a mean score for the following items: ease at falling asleep, sleep quality, calm sleep, and slept throughout the night (Åkerstedt et al., 1994). DOMS was evaluated by means of a Likert scale with six numbered levels of pain perception (Vickers, 2001).

HRV recordings were taken every morning upon awakening, after emptying the bladder, in the supine position, with eyes closed, spontaneous breathing, without making any movement during the recording. The recording had two minutes of stabilization, followed by six minutes of recording (Javaloyes et al., 2019). This was captured through the Elite HRV© application, available for smartphones with a compatible Polar H7 chest strap (Polar Electro, Kempele, Finland). Finally, the Kubios Standard© (v.3.5) software (Kubios Oy, University of Eastern Finland, Kuopio, Finland) was used for HRV analysis.

The following variables were evaluated: RR intervals (RR), root mean square of successive differences of RR intervals (RMSSD), the percentage of successive RR intervals that differed by more than 50 ms (pNN50), standard deviation of normal-to-normal bits (SDNN), the natural logarithm of high-frequency 0.15 to 0.4 Hz (HFln), a stress score (SS), sample entropy (SampEn), and the sympathetic-parasympathetic index (S/Ps) (Orellana et al., 2015; Shaffer and Ginsberg, 2017).

### Design and Procedures

A comparative and randomized cross-over experimental design was used to identify the delayed effects of three different resistance-training sessions 24 and 48 h into recovery on cardiac autonomic activity, sleep quality, and DOMS in physically trained individuals. Participants performed squat exercise on a Smith machine in three different training sessions based on movement velocity loss separated by one week. The velocity-based training methodology was used for monitoring all training sessions and tests (Cui et al., 2024). Before each training session, a 1RM 2 point-test based on movement velocity was performed to determine the 1RM (García-Ramos et al., 2018). All training sessions included four sets with three minutes of rest between sets, and these were performed at 60% of 1RM. Also, the warm-up consisted of joint mobility of the lower limbs: hip, knees, and ankles, 10'' each. There were also five minutes of cycloergometer pedaling at 50 W and 70 rpm, followed by three sets of five repetitions of squats with the weight of the bar, namely 30 kg, were included; and then two sets at a progressive concentric contraction velocity.

Each participant descended in a continuous motion until the upper thighs were below the horizontal plane, the posterior thighs and legs contacting each other. The feet were positioned just below the weight projection of the bar, with a straight back, and the gaze straight ahead. The maximum downward velocity was set at 0.50 and 0.70 m/s, while the upward velocity was maximum. Participants were not allowed to lift their heels off the floor or the trapeze bar at any time during the concentric phase of the movement; if this occurred, that execution was eliminated and repeated after three minutes. In each set, the repetition with the highest mean propulsive velocity (MPV) in the concentric phase was registered for further analysis. Three repetitions were performed with 30 kg and at least two repetitions with body mass. Passive recovery time between subsequent loads was established at three minutes.

On the other hand, and in order to calculate the training load, the greatest amount of weight that could be lifted with the correct technique (1RM) was identified, and this was considered as the load projected at the mean propulsive velocity, that is, 0.32 m/s (Sánchez-Medina et al., 2017) according to the linear regression equation (Loturco et al., 2016). Likewise, the bar movement velocity was calculated using a linear encoder (T-Force Dynamic Measurement System; Ergotech Consulting S. L., Murcia, Spain), and squat exercise was performed on a Smith machine (H. A. Bicicletas S. A., Sportfitness, Medellín, Colombia).

Finally, three training sessions were performed with different percentages of movement velocity loss, up to 10%, 20% and 40%. All sessions were performed at 60% of the 1RM calculated before using the 2-point method, and the training load was approximated to the nearest whole number. The sessions consisted of four sets, each ended when the participant exceeded the percentage of velocity loss allowed. The rest interval between sets was that of three minutes. At this point, it is necessary to point out that the investigator always supervised participants to control the compliance with the protocol.

### Statistical Analysis

The variables of HRV, SQI, DOMS and repetitions were analyzed with the SPSS statistical package V.24 (SPSS Inc., Chicago, IL, USA). This software was used to calculate mean and standard deviations. Normality analyses were performed with the Shapiro-Wilk test. To study the main effects and interactions between time points in each variable, we performed a one-way analysis of variance. The pair comparisons were carried out with the Bonferroni pos-hoc. A level of *p* ≤ 0.05 was set to indicate statistical significance. Eta squared partial (η^2^_p_) was calculated to assess the effect size of the comparisons. All the results are presented as mean ± standard deviation.

## Results

**Figure 2:**
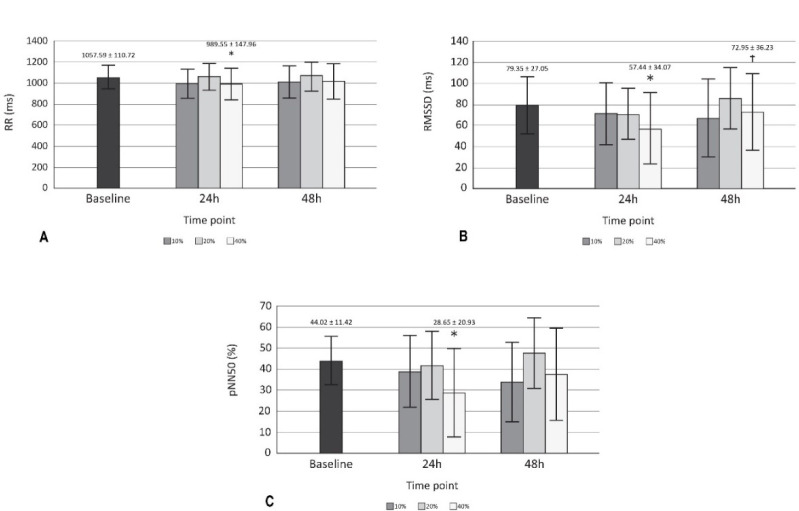
HRV variables with changes A) Comparison of RR among baseline, 24 and 48 h across each type of training; B) Comparison of RMSSD among baseline, 24 and 48 h across each type of training; C) Comparison of pNN50 among baseline, 24 and 48 h across each type of training. RR = RR intervals. * p < 0.05 compared to baseline; RMSSD = root mean square of successive RR interval differences. † p < 0.05 compared to 24 h 40%. pNN50 = percentage of successive RR intervals that differ by more than 50 ms

**Figure 3:**
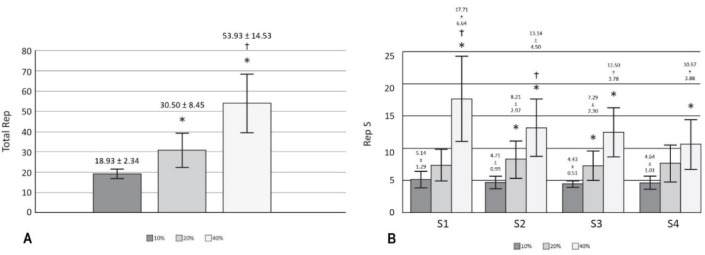
Training repetitions A) Comparison of total repetitions between each type of training; B) Comparison of repetitions per sets across each type of training. * p < 0.05 compared to 10%; † p < 0.05 compared to 20%

**Figure 4:**
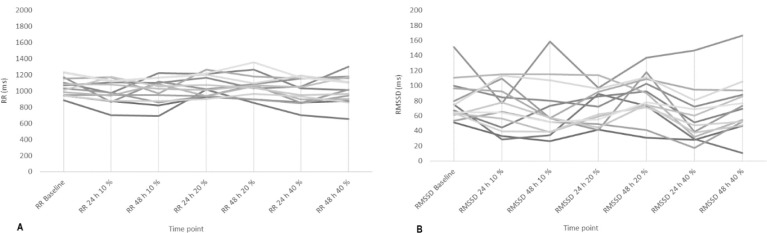
Intersubject variability of exercise response A) RR intervals; B) Root mean square of successive RR interval differences (RMSSD).

Significant effects (*p* < 0.05) on RR, RMSSD, pNN50 (Figure 2) and DOMS (Table 2) after 24 h of training with 40% of velocity movement loss were found, and the changes found were related to the baseline. Other variables calculated, such as SDNN, HFln, SS, SampEn and S/Ps (Table 1) did not show significant changes; the weight of the bar was maintained without relevant variations for the three training sessions performed (55.79 ± 13.62 kg for 10%, 54.64 ± 13.78 kg for 20%, and 53.93 ± 13.25 kg for 40%); and the number of total and set repetitions was significantly higher as the percentage of movement velocity loss increased (Figure 3). Considering a possible intersubject variability of exercise response, Figure 4 illustrates the differences among participants. Finally, no statistical differences were observed when comparing the number of repetitions in different sets at the same intensity (*p* > 0.05), and there were no changes in sleep quality between the baseline and 24 and 48 h post-training (Table 2).

**Table 1:** HRV variables unchanged.

Variable	Time point	Velocity loss (%)			ANOVA		
		10%	20%	40%	F	*p*	η^2^_p_
		Mean ± SD	Mean ± SD	Mean ± SD			
SDNN	Baseline	101.81 ± 32.53			1.363	0.334	0.505
	24 h	101.95 ± 35.13	96.97 ± 41.08	89.70 ± 47.44			
	48 h	93.42 ± 43.30	106.37 ± 33.79	106.78 ± 44.23			
HFln	Baseline	7.49 ± 0.66			4.894	0.022	0.786
	24 h	7.18 ± 1.00	7.25 ± 0.79	6.68 ± 1.23			
	48 h	7.06 ± 1.08	7.64 ± 0.92	7.01 ± 1.37			
HFnu	Baseline	46.57 ± 9.53			3.989	0.038	0.749
	24 h	33.62 ± 11.70^*^	44.50 ± 17.35	36.79 ± 18.76			
	48 h	42.03 ± 17.49	52.42 ± 19.68	41.39 ± 22.11			
SS	Baseline	9.12 ± 2.63			1.085	0.444	0.449
	24 h	8.46 ± 3.28	9.88 ± 4.88	10.58 ± 4.80			
	48 h	9.67 ± 3.65	8.36 ± 3.47	9.38 ± 6.37			
SampEn	Baseline	1.52 ± 0.16			1.246	0.376	0.483
	24 h	1.38 ± 0.20	1.53 ± 0.36	1.32 ± 0.35			
	48 h	1.36 ± 0.43	1.46 ± 0.32	1.29 ± 0.39			
S/Ps	Baseline	0.21 ± 0.11			3.471	0.054	0.722
	24 h	0.23 ± 0.20	0.24 ± 0.18	0.42 ± 0.41			
	48 h	0.27 ± 0.18	0.18 ± 0.18	0.41 ± 0.86			

SDNN = standard deviation of normal-to-normal bits. HFln = natural logarithm of high-frequency (0.15–0.4 Hz). HFnu = relative power of the high-frequency band (0.15–0.4 Hz) in normal units. SS = stress score. SampEn = sample entropy. S/Ps = sympathetic-parasympathetic index. * p < 0.05 compared to baseline; † p < 0.05 compared to 24 h 40%.

**Table 2:** Sleep quality index and delayed onset muscle soreness.

Variable	Time point	Velocity loss (%)			ANOVA		
		10%	20%	40%	F	*p*	η^2^_p_
		Mean ± SD	Mean ± SD	Mean ± SD			
SQI	Baseline	4.38 ± 0.47			0.343	0.896	0.204
	24 h	4.50 ± 0.48	4.52 ± 0.46	4.52 ± 0.49			
	48 h	4.39 ± 0.48	4.46 ± 0.47	4.48 ± 0.66			
DOMS	Baseline	0.12 ± 0.18			6.749	0.008	0.835
	24 h	1.86 ± 1.83	1.64 ± 1.65	2.86 ± 1.75^*^			
	48 h	1.64 ± 1.91	1.00 ± 1.18	2.64 ± 1.65^*^			

* p < 0.05 compared to baseline

## Discussion

The aim of the present investigation was to evaluate the delayed effects of velocity loss-based resistance training on HRV. To this end, fourteen men performed daily recordings of HRV, SQI and DOMS. Significant changes (*p* < 0.05) after 24 h of training were found in DOMS and HRV variables, specifically in RR, RMSSD, and pNN50, between 40% of velocity movement loss and the remaining conditions. The results show that training at 10% and 20% velocity loss did not significantly modify HRV as previously reported by Formalioni et al. (2020), where some training load components were modified (the percentage of 1RM, the number of sets and rest intervals between sets). However, it is important to emphasize that, in the present study, each workout consisted of only four sets of one exercise (squat), thus the total number of repetitions completed at 10% (18.93 ± 2.34) and 20% (30.50 ± 8.45) could have been insufficient to induce significant fatigue (Figure 3). The same explanation could refer to changes found in DOMS (Table 2).

In the same way, it has been proposed that the increase in volume would affect RMSSD (Marasingha-Arachchige et al., 2020); therefore, it is necessary to explain that training with the highest number of repetitions (40%: 53.93 ± 14.53) was more likely to have generated significant changes in this variable in contrast to the other two sessions. RMSSD has already been tested to prescribe the recovery interval for resistance training, and a higher weekly frequency of sessions was found to maintain neuromuscular adaptations (De Oliveira et al., 2019). Intense resistance training has been shown to affect the natural logarithm of RMSSD immediately afterwards; however, 24 h later, the value is re-established, and the standing measurement appears to be more co-relatable with neuromuscular and perceptual markers of recovery (Flatt et al., 2019). Similarly, a dosage according to the character of the effort would be useful. Pareja-Blanco et al. (2017) previously compared two types of effort (total effort and half effort), and their results showed that total effort significantly affected HRV recovery after six hours.

Regarding the recovery process, it has been reported that vascular occlusion exercises, also known as Kaatsu training, show a shorter autonomic recovery time than conventional training (Okuno et al., 2014). Also, dynamic resistance exercise demonstrates greater vagal modulation compared to static exercise (Roy et al., 2020), which may cause a faster recovery compared to static exercise. In short, different types of resistance training strategies can affect the autonomic response measured by HRV, making evident marked sensitivity to changes.

On the other hand, the effects of resistance training to muscle failure 24 h after its completion have been previously studied, and, although a significant decrease in 1RM was observed, no changes in nocturnal HRV or sleep quality were noted (Ramos-Campo et al., 2021). The protocols used by Ramos-Campo et al. (2021) consisted of 4 sets of 10 repetitions to failure, and 5 sets of 10 repetitions with 2 reserve repetitions. Moreover, both workouts used two exercises, one for the upper and one for the lower body, at 75% of 1RM and adding 40 repetitions per exercise. It was found that only the session of 53.93 ± 14.53 repetitions led to decreased HRV. However, no baseline period was considered to determine an average behavior of the ANS. In our study, we included such a period in order to minimize the risk of the high daily variability of HRV.

It was also reported that high and low frequency values returned to their baseline levels 45 min after resistance training sessions consisting of four exercises, three sets each, at 15RM, regardless of the rest times used (Alemi et al., 2022); for this case the high frequency also did not show changes under any type of a training session. This finding contrasts with another study where a two-week intense resistance training protocol, involving six hypertrophic exercises per session, affected nocturnal RMSSD and high-frequency power, averaged over three subsequent nights. However, changes in HRV were found during a single session with velocity-based resistance training that allowed for a higher velocity loss threshold, leading to more repetitions per set (Kaikkonen et al., 2020).

In the same way, a high correlation of SS and S/Ps with the rate of perceived exertion in aerobic exercise has been previously reported (Abellán-Aynés et al., 2019); however, in the present study, these indices did not show significant changes after training sessions, yet DOMS was altered 24 h after training at 40% loss of velocity. This may question the usefulness of SS and S/Ps as indicators of internal loading in resistance training.

In the literature, other conditions have been also reported concerning resistance training and HRV. It has been described that the autonomic cardiac response induced by resistance exercise could be conditioned considering the amount of muscle mass involved in the training session, being more intense with a higher mass involved. This effect lasted for more than 30 min, and the probable reason was the greater muscle mass in the lower limbs, but it is not clear whether the recovery was similar or not (Isidoro et al., 2017). Regarding the type of exercise, it is important to mention the results presented by Álvarez-Herms et al. (2020), where a protocol of six sets of 15-s continuous jumps performed at three different simulated altitudes did not alter HRV indicators 24 h later. Therefore, efforts that last more than 20 min are probably required to achieve an alteration in autonomic homeostasis.

Finally, as a physiological hypothesis, we believe that it is likely that stimulation of the c-fibers (nociceptors) (Borghi et al., 2022) causes an increase in the sympathetic afferent impulse, which would lead to an increase in chronotropism, and therefore a decrease in HRV. This theory is based on the fact that HRV is affected by the behavior of c-fibers (Forstenpointner et al., 2020), in this case, perhaps by a mechanism very similar to the viscerosomatic inhibition by exercise that occurs in the lungs (Gandevia et al., 2000).

There are some limitations of this study which should be acknowledged. Although our study participants were physically active, sports students, their experience with resistance training was diverse, which could have influenced post-training responses. Likewise, although training, familiarization and recommendations were provided, HRV records were taken by the participants themselves. As future lines of research in this area, we propose to evaluate the effects between the upper and the lower body to clarify possible effects by muscle groups and to experiment with different monitoring protocols and adjustment of the load according to the behavior of the muscles.

## Conclusions

The present study concludes that resistance training with greater losses of movement velocity may result in greater internal load stimuli, according to the autonomic modulation, as measured by HRV. Likewise, RR, RMSSD and pNN50 indicators seem to be most sensitive 24 h after a resistance training session. In the same way, it can be evidenced that using the 2-point method to estimate the 1RM before training is a useful and practical tool that allows adjusting the training load to the state prior to the effort. Similarly, there are significant differences in the number of total repetitions between the resistance training strategies according to the loss of execution velocity (10%, 20% and 40%). The strategy of 40% velocity loss generated a relevant change in DOMS, and sleep quality was not affected by any of the training sessions.
